# *In Vivo* Multimodal Imaging of Drusenoid Lesions in Rhesus Macaques

**DOI:** 10.1038/s41598-017-14715-z

**Published:** 2017-11-03

**Authors:** Glenn Yiu, Eric Tieu, Christian Munevar, Brittany Wong, David Cunefare, Sina Farsiu, Laura Garzel, Jeffrey Roberts, Sara M. Thomasy

**Affiliations:** 1 Department of Ophthalmology & Vision Science, University of California, Davis, Sacramento, California, USA; 2 Department of Surgical and Radiological Sciences, School of Veterinary Medicine, University of California, Davis, Davis, California, USA; 3 Department of Biomedical Engineering, Duke University, Durham, North Carolina USA; 4 California National Primate Research Center, Davis, California, USA

## Abstract

Nonhuman primates are the only mammals to possess a true macula similar to humans, and spontaneously develop drusenoid lesions which are hallmarks of age-related macular degeneration (AMD). Prior studies demonstrated similarities between human and nonhuman primate drusen based on clinical appearance and histopathology. Here, we employed fundus photography, spectral domain optical coherence tomography (SD-OCT), fundus autofluorescence (FAF), and infrared reflectance (IR) to characterize drusenoid lesions in aged rhesus macaques. Of 65 animals evaluated, we identified lesions in 20 animals (30.7%). Using the Age-Related Eye Disease Study 2 (AREDS2) grading system and multimodal imaging, we identified two distinct drusen phenotypes – 1) soft drusen that are larger and appear as hyperreflective deposits between the retinal pigment epithelium (RPE) and Bruch’s membrane on SD-OCT, and 2) hard, punctate lesions that are smaller and undetectable on SD-OCT. Both exhibit variable FAF intensities and are poorly visualized on IR. Eyes with drusen exhibited a slightly thicker RPE compared with control eyes (+3.4 μm, P=0.012). Genetic polymorphisms associated with drusenoid lesions in rhesus monkeys in ARMS2 and HTRA1 were similar in frequency between the two phenotypes. These results refine our understanding of drusen development, and provide insight into the absence of advanced AMD in nonhuman primates.

## Introduction

In humans, drusen are focal deposits of extracellular debris located between the retinal pigment epithelium (RPE) and Bruch’s membrane, and may occur with normal aging or as a sign of age-related macular degeneration (AMD) - the leading cause of vision loss in the elderly. Drusen are primarily composed of lipids such as phosphatidylcholine, esterified cholesterol, and unesterified cholesterols^1–3^, as well as various proteins including vitronectin, crystallins, amyloid proteins, apolipoproteins, immunoglobulins, and complement components^4–6^. The advent of ocular imaging technologies has led to more refined classification of different drusen phenotypes, including soft drusen, cuticular drusen, and reticular pseudodrusen. Soft drusen are larger, yellow-white mounds that are histologically external to the RPE basement membrane. Cuticular drusen are smaller, densely-packed, and more uniform in size. They were previously thought to be nodular thickenings of the RPE basement membrane^7^, but were subsequently found to be external to the basal lamina of the RPE with a similar composition as soft drusen^8^. Reticular pseudodrusen are located in the subretinal space above the RPE^9,10^, and may be distinguished by their location on histology and optical coherence tomography (OCT)^11^. These different drusen phenotypes are associated with certain genotypic variants such as those in the complement factor H (CFH) and age-related maculopathy susceptibility (ARMS2) loci, and may have differential propensity for progression to RPE atrophy and late-stage AMD^12,13^.

Most laboratory animals such as rodents are poor models of AMD and drusen due to their relative short lifespan and lack of a macula. In contrast, nonhuman primates are the only mammals that possess a true macula comparable to humans, and spontaneously develop drusenoid lesions similar to those seen in AMD. Early studies reported a high prevalence of drusenoid deposits in free-ranging colonies of rhesus macaques (*Macaca mulatta*) from Cayo Santiago in Puerto Rico, where drusenoid pathology was noted in 30–74% of animals^14–17^. Subsequent studies of other colonies within the continental U.S. demonstrated a lower prevalence of 5–47%^18–20^, likely due to factors such as reduced sunlight exposure from indoor housing, more regular diet, age and genetic differences between colonies, as well as different methods of detecting and classifying the lesions.

Prior studies of drusenoid deposits in nonhuman primates were mostly qualitative, relying on clinical examination, fundus photography, and histological analysis to characterize the lesions. The deposits were classified as “hard” or “soft” based on funduscopic appearance, followed by post-mortem assessment of their histopathology. These studies revealed increasing frequency of drusenoid deposits with age^14,21^, and similar molecular composition as human drusen^22^. Rhesus monkeys and humans were also found to share genetic polymorphisms in genes such as ARMS2 and HTRA1 (high temperature-requirement factor A1) that are associated with drusen formation^23–25^. These studies suggest that the drusenoid deposits in nonhuman primates may offer significant insight into drusen formation in humans, but more refined, quantitative methods are necessary to characterize these lesions in monkeys for translational research.

In this study, we surveyed aged rhesus macaques (age >19 years) at the California National Primate Research Center (CNPRC) to identify animals with drusenoid lesions. We used the validated age-related eye disease study 2 (AREDS2) system and digital image segmentation for grading drusen from fundus photographs in human studies^26,27^, then employed *in vivo* ocular imaging modalities, including blue-peak fundus autofluorescence (FAF), infrared reflectance (IR), and spectral domain-OCT (SD-OCT), to characterize the drusenoid lesions in rhesus macaques *in vivo*. We identified two morphologic phenotypes of drusenoid deposits, which can be distinguished by different imaging features, and investigated potential associations between drusen phenotype with single-nucleotide polymorphisms in ARMS2 and HTRA1 which are known to be associated with macular drusen in rhesus monkeys. Comparing our results with prior studies of drusen prevalence in other primate colonies, we speculate on the pathogenesis of drusen formation in rhesus macaques, and the absence of advanced AMD in nonhuman primates.

## Methods

### Subjects

The CNPRC is accredited by the Association for Assessment and Accreditation of Laboratory Animal Care (AAALAC) International. All studies using rhesus macaques (*Macaca mulatta*) followed the guidelines of the Association for Research in Vision and Ophthalmology Statement for the Use of Animals in Ophthalmic and Vision Research, complied with the National Institutes of Health (NIH) Guide for the Care and Use of Laboratory Animals, and were approved by the University of California, Davis Institutional Animal Care and Use Committee. Complete ophthalmic examinations were performed in aged rhesus macaques (≥ 19 years) undergoing routine semi-annual physical examinations. The animals were sedated with intramuscular injection of ketamine hydrochloride, midazolam, and dexmedetomidine, followed by pupillary dilation with tropicamide (Bausch & Lomb, Tampa, FL) and cycloplegia with cyclopentolate (Akorn, Lake Forest, IL). All subjects underwent slit lamp biomicroscopy, dilated fundus biomicroscopy, streak retinoscopy, A-scan biometry, and rebound tonometry. After the initial survey, multimodal imaging of the macula was performed on all animals with drusenoid lesions identified on fundus examination, as well as a selection of age-matched control animals, including fundus photography, blue-peak FAF, IR imaging, and SD-OCT. Animal eyes that showed any other retinal or choroidal lesions on exam were excluded from this study. For all animals, age, sex, cycloplegic refraction (diopters), intraocular pressure (mmHg) measured by rebound tonometry, and axial length (mm) on A-scan biometry were recorded.

### Multimodal Ocular Imaging

Fundus photography was performed using the CF-1 Retinal Camera (Canon, Tokyo, Japan) with a 50-degree wide-angle lens. Blue-peak FAF and IR imaging were obtained simultaneously with SD-OCT using the Spectralis HRA+OCT platform (Heidelberg Engineering, Heidelberg, Germany). Confocal scanning laser ophthalmoscopy was used to capture 30º × 30º autofluorescence images using an excitation light of 488 nm and a long-pass barrier filter starting at 500 nm, along with a 20 º × 20º SD-OCT volume scan with 1024 A-scans per B-scan and raster line spacing of 51 μm, centered on the fovea, in high-resolution mode. Infrared reflectance images were captured using an 820 nm diode laser, along with a 30 º × 5º SD-OCT raster scan with 1536 A-scans per B-scan and 234 μm spacing between B-scans, in high-resolution enhanced-depth imaging (EDI) mode. 25 scans were averaged for each B-scan, using the Heidelberg eye tracking Automatic Real-Time (ART) software. All imaging was performed by the first author (GY) at CNPRC. Animals were monitored by a trained technician and a CNPRC veterinarian (LG) at all times.

### Image Analysis

**Figure 1:**
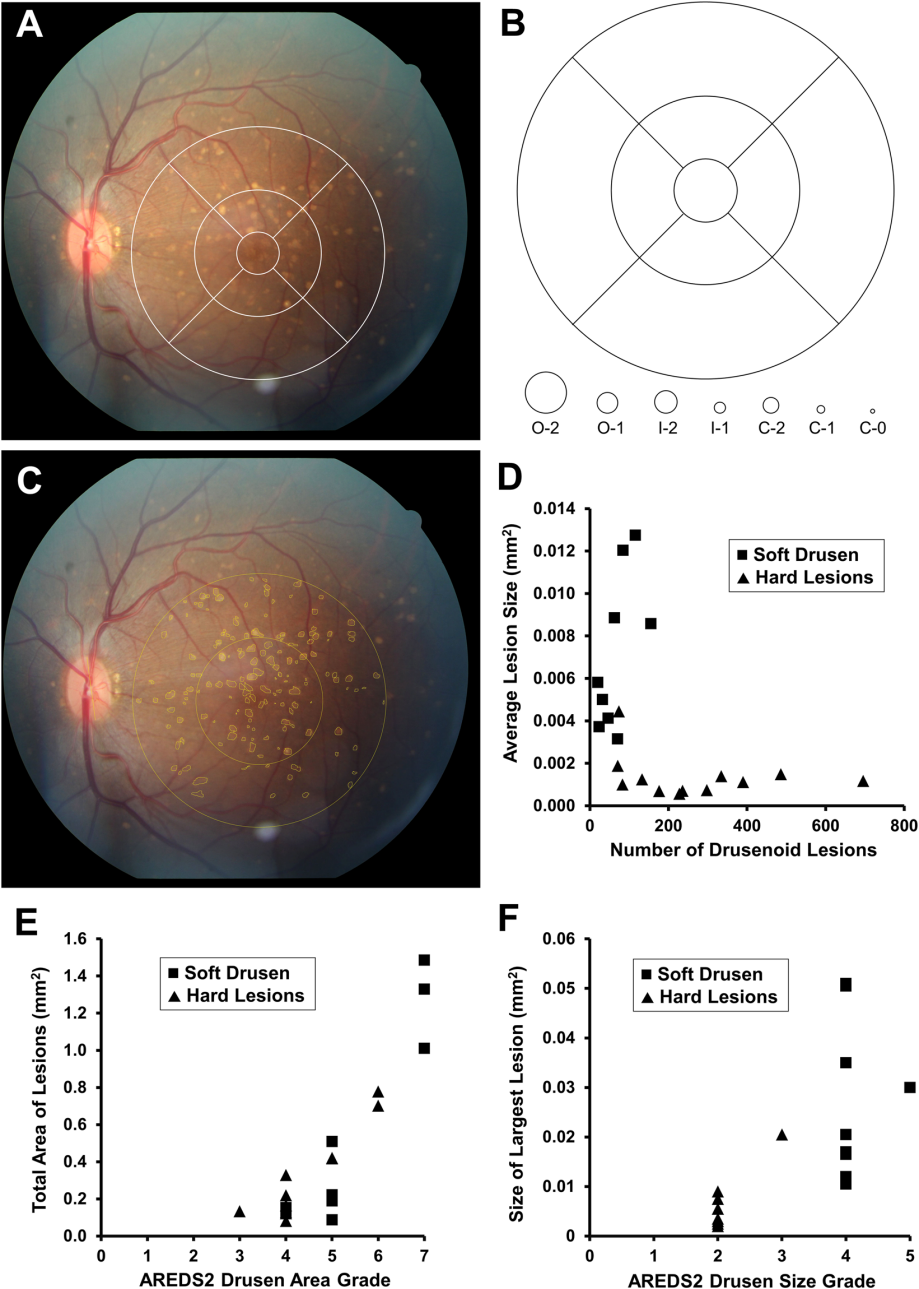
Grading and quantification of drusenoid lesions in rhesus macaques from fundus photographs. A representative fundus photograph of a left rhesus macaque eye with large soft drusen is shown overlaid with the modified Early Treatment Diabetic Retinopathy Study (ETDRS) grid template (**A**), along with a diagram of the graduated measurement circles used for estimating the drusen size and area based on a modified Age-Related Eye Disease Study 2 (AREDS2) grading system (**B**). Both the grid template and measurement circles are calibrated to the vertical dimension of the rhesus optic nerve, which is 1400 μm in adult animals (compared to 1800 μm in human adults). The same representative fundus photograph is shown with manual segmentation of drusenoid lesions (yellow outlines), as well as the inner and outer rings (yellow circles) (**C**). Scatterplots show the inverse relationship between number of drusenoid lesions and average lesion size (**D**), as well as the association between AREDS2 drusen area grade with total segmented lesion area (**E**) and between AREDS2 drusen size grade with largest segmented lesion size (**F**).

Digital color fundus photographs were evaluated by two experienced image graders (GY, ET) using ImageJ (version 1.49v; National Institutes of Health, Bethesda, MD). Drusenoid lesions were categorized using the Age-Related Eye Disease Study 2 (AREDS2) system for classification of AMD, which has been previously validated for grading of digital fundus photographs in human clinical trials with high reproducibility^26^. Grading involves a modified grid template adapted from the Early Treatment Diabetic Retinopathy Study (ETDRS)^28^, with a set of graduated circles used to estimate maximum drusen size and total area involved by pigment abnormalities and drusen (Fig. 1A). The grid template was calibrated in these animals based on the ocular dimensions of the optic disc in rhesus macaques, which measures 1400 μm vertically and 1000 μm horizontally^29^, compared to 1800 μm in both dimensions in humans^26^. The radii of the modified grid template circles remain as 1/3 DD, 1 DD, and 2 DD based on the vertical disc diameter, but are equivalent to 467 μm, 1400 μm, and 2800 μm respectively (Fig. 1B). Rhesus and human studies can be compared by applying a scaling constant of 0.778 for linear measurements, and 0.605 for area measurements. Lesions graded include area of hyperpigmentation or hypopigmentation; maximum size and area of drusen within the grid; presence of calcified drusen, drusenoid pigment epithelial detachment, or reticular drusen; presence, area, configuration, and center-involvement of geographic RPE atrophy; and presence of neovascular AMD (Supplemental Table 1). The details of the grading criteria have been extensively described^26,30^. Any discrepancies in grading were resolved by open adjudication between the graders. Manual drusen segmentation of the fundus photographs was then performed by the two graders by outlining individual lesions within the inner and outer ETDRS circle using the freehand selection tool in ImageJ as previously described^27^ and calibrated to the vertical diameter of the optic disc. All measurements were determined from the mean of the two graders’ measurements, and include the number of drusenoid lesions, total area of lesions, average lesion size, and size of largest lesion (Fig. 1C).

**Table 1:** Fundus photograph grading of drusenoid lesions in Rhesus macaques using the AREDS2 classification system.

	Any Drusen (n = 40)	Soft Drusen (n = 16)	Hard Lesions (n = 18)	P-value
**Drusen size** (largest)				<0.001*
0: None	0	0	0	
1: Questionable	0	0	0	
2: Definite, diameter < C-0^&^	17	0	17	
3: Diameter > C-0, but < C-1	1	0	1	
4: Diameter > C-1, but < C-2	15	15	0	
5: Diameter > C-2	1	1	0	
8: Cannot grade	6	0	0	
**Drusen area** ^#^				0.102
0: Questionable, or total area < C-0	0	0	0	
1: Area ≥ C-0, but < C-1	0	0	0	
2: Area ≥ C-1, but < C-2	2	0	2	
3: Area ≥ C-2, but < I-2	4	1	3	
4: Area ≥ I-2, but < O-2	16	7	9	
5: Area ≥ O-2, but < ½ DA	7	3	4	
6: Area ≥ ½ DA, but < 1DA	2	1	1	
7: Area ≥ 1DA	3	3	0	
8: Cannot grade	6	0	0	
**Drusen outside grid**				cannot compute
0: None, questionable, or area < O-2	33	15	18	
5: Area ≥ O-2	1	1	0	
8: Cannot grade	6	0	0	

^*^Statistically significant.

^#^Area within modified Early Treatment of Diabetic Retinopathy Study (ETDRS) grid.

^&^The circular areas for grading measured in square millimeters are: C-0 = 0.003 mm^2^, C-1 = 0.011 mm^2^, C-2 = 0.043 mm^2^, I-2 = 0.089 mm^2^, O-2 = 0.297 mm^2^, 1/2 disc area (DA) = 0.77 mm^2^, 1 DA = 1.54 mm^2^.

The SD-OCT images were exported from Heidelberg Explorer software (version 1.8.6.0, Heidelberg Engineering, Heidelberg, Germany) to Duke Optical Coherence Tomography Retinal Analysis Program (DOCTRAP, version 62.0)^31,32^, a custom image segmentation software designed using MATLAB (Mathworks, Natick, MA). For thickness measurements of retinal and choroidal layers, segmentation boundaries were automatically determined by DOCTRAP, followed by manual refinement by two experienced graders (CM, BW) along a 3 mm horizontal segment centered on the fovea. Measured layers include nerve fiber layer (NFL), ganglion cell/inner plexiform layers (GCL/IPL), inner nuclear layer (INL), outer plexiform layer (OPL), outer nuclear layer (ONL), photoreceptor inner and outer segments (IS/OS), retinal pigment epithelium (RPE) which includes any drusenoid complex between the RPE and Bruch’s membrane, as well as the choriocapillaris (CC), and outer choroid (OC). The GCL/IPL were measured as a single complex due to difficulty in distinguishing between these layers even in normal retinal scans. The total retinal thickness was measured from the internal limiting membrane to Bruch’s membrane, and total choroidal thickness was measured from Bruch’s membrane to the choroidal-scleral junction as previously described^33,34^. Measurements were determined from the mean of the two graders’ measurements, and then averaged across the central 3mm for thickness comparisons.

Correlation in drusen size and area between left and right eyes was determined using two-tailed Pearson correlation. Differences in chorioretinal layer thickness measurements and differences in lesion number, size, and area were compared using linear regression analysis with generalized estimating equations to account for two eyes evaluated from each animal. The association of AREDS2 grading with segmented measurements was also determined using linear regression with generalized estimating equations.

### Genotype Analysis

Genomic DNA from blood or buffy coat was extracted with a commercial kit (DNeasy Blood and Tissue Kit; Qiagen, Hilden, Germany) following the manufacturer’s protocol, and quantified using a spectrophotometer (NanoDrop 2000c; Thermo Fisher Scientific, Waltham, MA, USA). The region of interest was amplified using the following primers HTRA1-Forward 5′-TATCACTTCACTGTGGGTCCGG-3′, HTRA1-Reverse 5′-GCGATTCGCGTCCTTCAAACTA-3′, ARMS2-Exon1-Forward 5′-GATGGCAGCTGGCTTGGCAAGG, ARMS2-Exon1-Reverse 5′-GGGGTAAGGCCTGATCATCTGCA-3′ with a high-fidelity DNA polymerase (Phusion or Q5; New England Biolabs, Ipswich, MA, USA) and purified using a PCR purification kit (QIAquick; Qiagen). Sanger sequencing of PCR products was performed by the University of California, Davis DNA Sequencing facility (Davis, CA, USA). Association of single nucleotide polymorphisms with drusen phenotype was determined using a Fisher’s Exact test. All statistical analyses were performed using SPSS Statistics (version 22, IBM).

## Results

### Demographics and Ocular Characteristics

Of 65 aged macaques (>19 years) evaluated at CNPRC, we identified drusenoid lesions by dilated fundus biomicroscopy in both eyes of 20 animals (30.7%). Of these animals, 10 were male, and mean age was 23.3 ± 2.7 years (range 19.3 to 29.4 years). Among the 40 eyes with drusenoid lesions, mean intraocular pressure (IOP) was 19.1 ± 4.4 mmHg, and mean axial length was 19.8 ± 0.7 mm. We also selected 8 age-matched animals with no ocular findings as normal controls for ocular imaging, with a mean age of 23.9 ± 2.9 years (range 21.3 to 28.2 years), of which 3 were male. Among the 16 control eyes, mean IOP and axial length were 17.1 ± 4.5 mmHg and 19.7 ± 1.0 mm, respectively. No statistical differences in age (P = 0.609), sex (P = 0.401), IOP (P = 0.298), and axial length (P = 0.945) were observed between the two groups of animals.

### AREDS2 Classification of Drusenoid Lesions on Fundus Photography

Among the 40 eyes noted to have drusenoid lesions on fundus examination, 34 eyes had fundus photographs with adequate quality for grading of drusenoid lesions (Table 1). Using the AREDS2 classification for drusen size, the majority of lesions were classified as “small” (grade 2, ≤59 μm diameter) or “large” (grade 4, >116 μm diameter), with similar proportions of each. A majority of eyes with drusenoid lesions had a total lesion area between grading circles I-2 (0.089 mm^2^) and O-2 (0.297 mm^2^). Left and right eyes of individual animals showed a strong correlation in drusen size (r = 0.989; P < 0.001) and area (r = 0.970; P < 0.001). No cases of calcified drusen, drusenoid pigment epithelial detachment, or reticular drusen were identified in any of the fundus photographs. There was also no evidence of hyperpigmentation, hypopigmentation, geographic atrophy, or neovascular AMD noted in any eye in this cohort of macaques.

We noted that drusenoid lesions in rhesus macaques adapted either a “soft” drusen or “hard” punctate appearance, based on the original AREDS terminology for drusen type. When classified in this manner, eyes with predominantly soft drusen showed significantly larger drusen size (P < 0.001) and a trend toward greater total lesion area (P = 0.102; Table 1). Some eyes with soft drusen also showed hard punctate lesions, while eyes with predominantly hard lesions rarely showed any soft drusen. Fellow eyes showed perfect correlation (r = 1.000; P < 0.001) in predominant lesion phenotype classified in this manner. Lesions were occasionally noted outside of the central macula, although only one animal exhibited any significant drusen (area ≥ 0.297 mm^2^) beyond the 5.6mm outer circle of the ETDRS grid (Table 1).

### Quantification of Drusenoid Lesions on Fundus Photography

Of eyes with digital fundus photographs, 21 were found to be adequate for image segmentation of individual drusenoid lesions. Quantitative drusen segmentation showed an inverse association between lesion size and number of lesions (Fig. 1D). Eyes with primarily soft drusen showed fewer number of lesions (P = 0.006) and larger average lesion size (P < 0.001), with a trend toward greater total lesion area (P = 0.297; Table 2). AREDS2 drusen area and size grading correlated well with the total lesion area and size of largest lesion as measured from image segmentation (P < 0.001 for both) (Fig. 1E,F). Hence, both AREDS2 grading and quantitative measurements from drusen segmentation demonstrate the distinct size and distribution of these two lesion phenotypes.

**Table 2:** Fundus photograph quantification of drusenoid lesions in Rhesus macaques using digital image segmentation.

	Any Drusen (n = 21)	Soft Drusen (n = 9)	Hard Lesions (n = 12)	P-value
Number of Lesions	181.5 ± 174.7^#^	67.7 ± 44.9	266.9 ± 188.4	0.006*
Total Area of Lesions (mm^2^)	0.418 ± 0.414	0.568 ± 0.558	0.305 ± 0.233	0.297
Average Lesion Size (mm^2^)	0.004 ± 0.004	0.007 ± 0.004	0.001 ± 0.001	<0.001*
Size of Largest Lesion (mm^2^)	0.015 ± 0.015	0.027 ± 0.016	0.006 ± 0.005	0.001*

^*^Statistically significant.

^#^All values expressed as mean ± standard deviation.

### Comparison of Drusenoid Lesions on OCT and FAF

**Figure 2:**
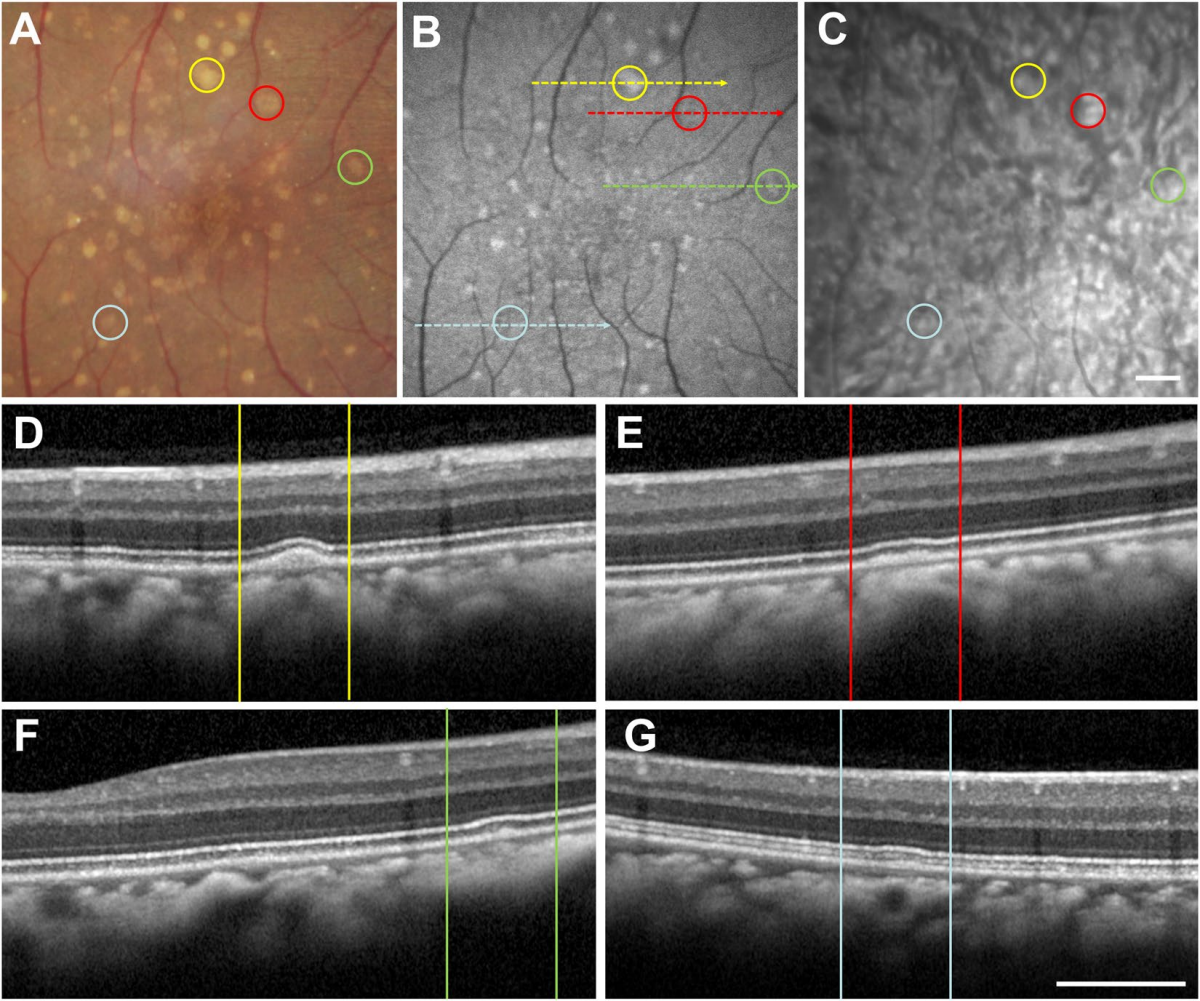
Multimodal imaging of soft drusen in rhesus macaques. Fundus photographs (**A**), as well as fundus autofluorescence (**B**), infrared reflectance (**C**), and spectral-domain optical coherence tomography (**D**–**G**) images of a right eye of a rhesus macaque with large soft drusen are shown, with corresponding drusen labeled with yellow, red, green, and blue circles (**A**–**C**) and lines (**D**–**G**). The colored dashed lines in (**B**) correspond to the line-scan segment shown in (**D**–**G**). Scale bars: 500 μm.

Image analysis of soft and hard lesions showed distinct characteristics on multimodal ocular imaging. Soft drusen (Fig. 2A) have a variable appearance on FAF imaging, with some lesions appearing more hyper-autofluorescent, and others having a more iso-autofluorescent appearance (Fig. 2B). Near IR imaging of the ocular fundus in rhesus macaques showed a mottled appearance corresponding to the underlying choroidal vascular architecture, and the drusenoid deposits are difficult to distinguish (Fig. 2C). On SD-OCT, the soft deposits appear as dome-shaped hyperreflective deposits located between the RPE and Bruch’s membrane (Fig. 2D–G). Deposits show variable autofluorescence intensities, with some larger lesions appearing more hyper-autofluorescent (Fig. 2D), while some less elevated ones show a less intense autofluorescence signature and may appear as focal thickening of the RPE itself (Fig. 2E–G). There was no clear disruption of the outer retinal layers, such as the external limiting membrane (ELM) or inner segment-outer segment (IS-OS) junction; or changes to the underlying choriocapillaris, which appear in rhesus macaques as a distinct hyporeflective band as our group has described recently^35^. The cone outer-segment tip (COST) lines, also known as the interdigitation zone, were variably visible in different animals, and showed no consistent difference between normal subjects and those with either drusen phenotype.

**Figure 3:**
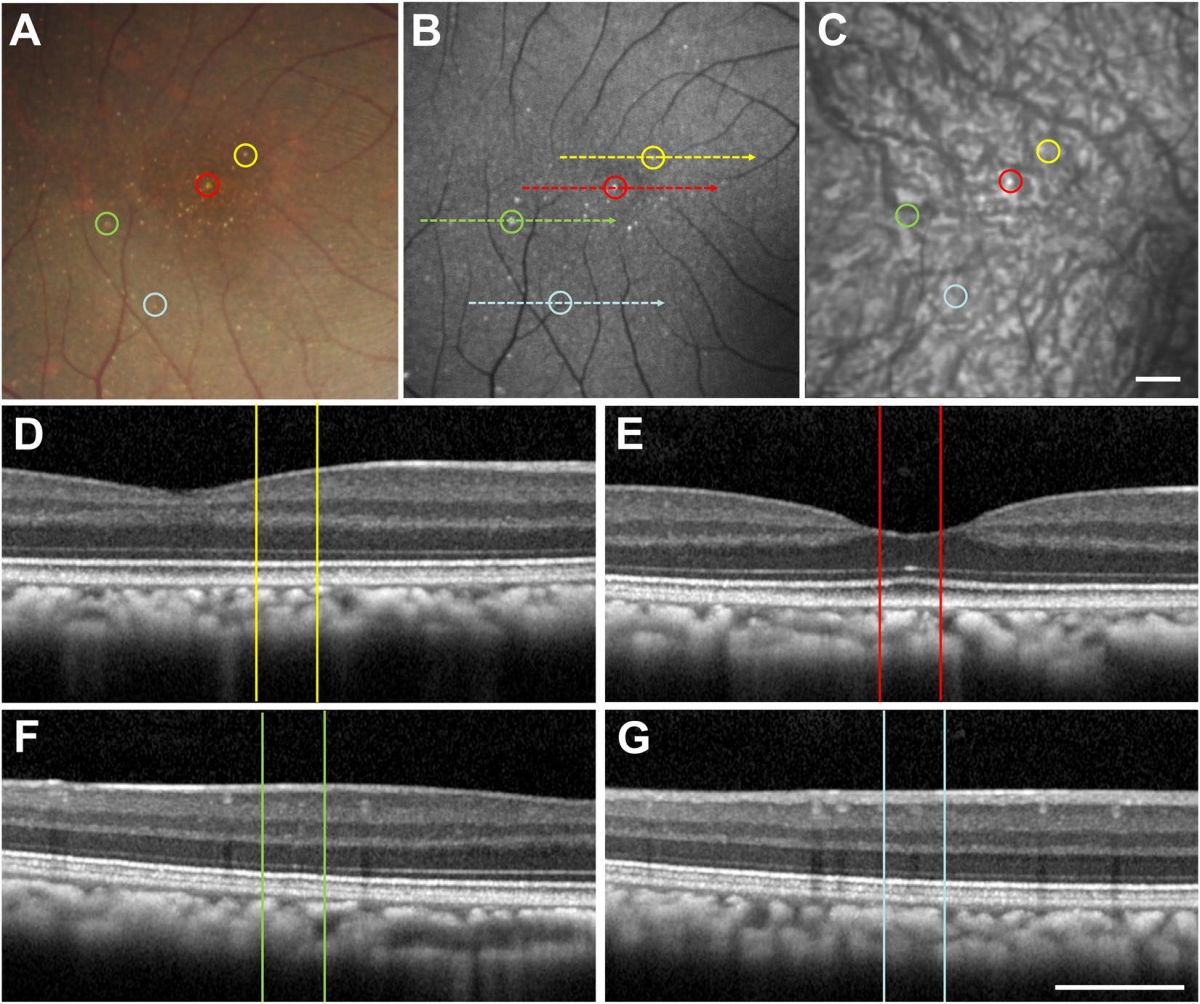
Multimodal imaging of hard punctate lesions in rhesus macaques. Fundus photographs (**A**), as well as fundus autofluorescence (**B**), infrared reflectance (**C**), and spectral-domain optical coherence tomography (**D**–**G**) images of a right eye of a rhesus macaque with hard, punctate lesions are shown, with corresponding lesions labeled with yellow, red, green, and blue circles (**A**–**C**) and lines (**D**–**G**). The colored dashed lines in (**B**) correspond to the line-scan segment shown in (**D**–**G**). Scale bars: 500 μm.

Hard punctate lesions (Fig. 3A) also have a variable appearance on FAF imaging, with some lesions appearing more hyper-autofluorescent while others are not as readily discernable (Fig. 3B). Rarely, these lesions may appear on near IR imaging (Fig. 3C), but most of the lesions could not be distinguished by this modality. Interestingly, the small punctate lesions were uniformly undetectable on even high density SD-OCT scans, with no visible disruptions of the RPE, outer retinal layers, or choroidal vasculature (Fig. 3D–G). Hence, these hard lesions do not appear to represent true sub-RPE deposits as in AMD drusen.

### Retinal and Choroidal Layer Thicknesses

**Figure 4:**
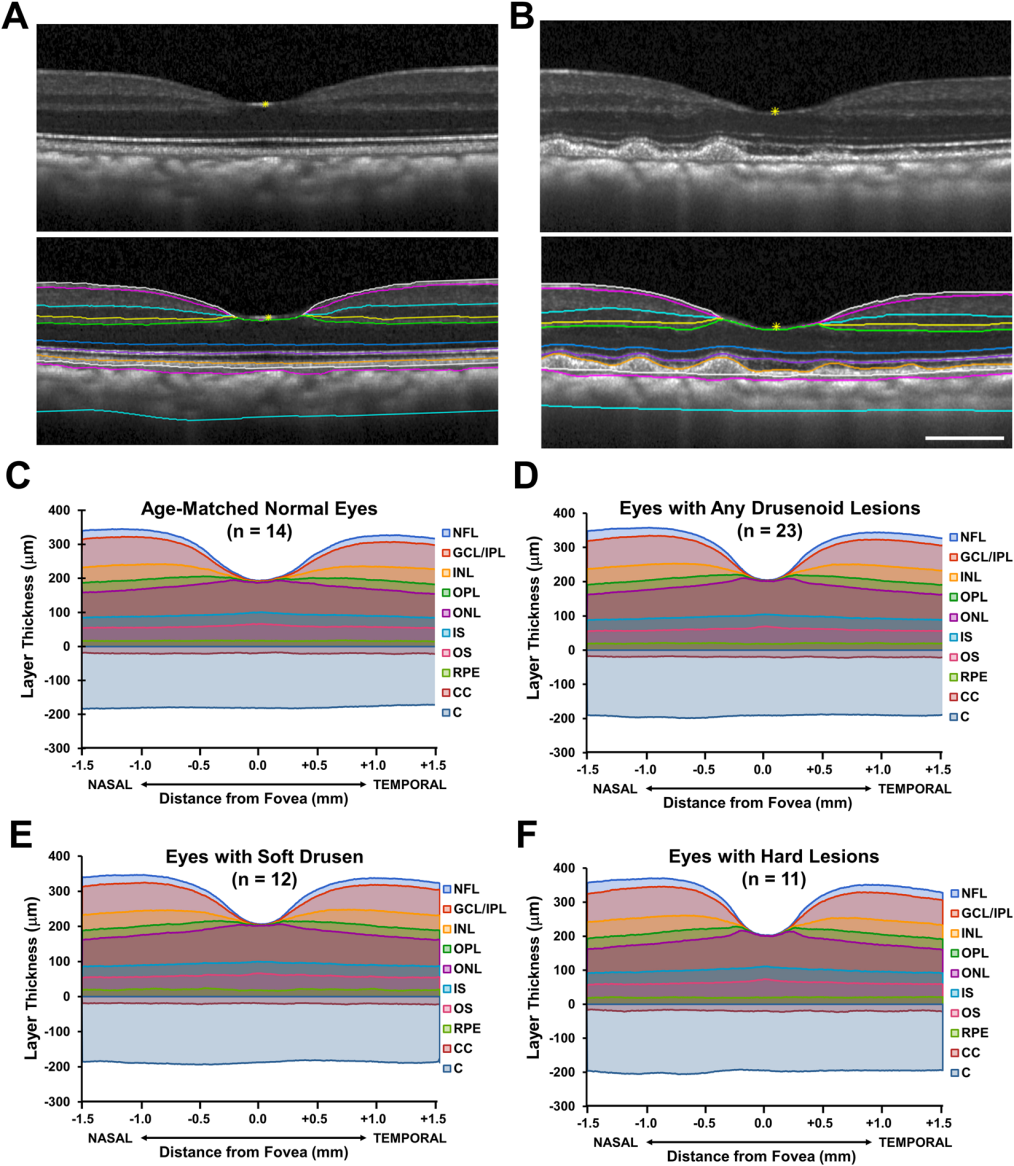
Image segmentation and thickness measurement of retinal and choroidal layers in rhesus macaques. Spectral-domain optical coherence tomography images from a normal age-matched eye (**A**) and an eye with soft drusen (**B**), with and without segmentation lines, are shown. Thickness of individual retinal and choroidal layers are shown for age-matched normal eyes (**C**), eyes with any drusenoid lesions (**D**), eyes with predominantly soft drusen (**E**), and eyes with hard, punctate lesions (**F**). Abbreviations: NFL, nerve fiber layer; GCL/IPL, ganglion cell/inner plexiform layers; INL, inner nuclear layer; OPL, outer plexiform layer; ONL, outer nuclear layer; IS, inner segments of photoreceptors; OS, outer segments of photoreceptors; RPE, retinal pigment epithelium; CC, choriocapillaris; C, outer choroid. Scale bar: 500 μm.

To compare retinal and choroidal thicknesses, we employed a custom, semi-automated segmentation software to measure the average thicknesses of individual layers across the central 3mm along a horizontal SD-OCT line scan through the fovea (Fig. 4A–B). Among the animals, 23 eyes with drusen and 14 eyes from age-matched controls had SD-OCT images of adequate quality for thickness measurements. Among these eyes, total retinal and choroidal thickness, as well as the thicknesses of individual layers, were similar between the two groups (Fig. 4C–D), with the exception of the RPE which was statistically thicker in eyes with drusenoid lesions by approximately 3.4 μm (P = 0.012; Table 3). When comparing eyes with predominantly soft drusen versus hard punctate lesions (Fig. 4E,F), there were no significant differences in any of the retinal or choroidal layers (Table 3).

**Table 3:** Retinal and choroidal layer thicknesses of age-matched normal eyes and eyes with drusenoid lesions in rhesus macaques.

	Normal Eyes (n = 14)	Any Drusen (n = 23)	P-value
Total retinal thickness	292.6 ± 21.4	314.0 ± 69.7	0.198
Nerve fiber layer (NFL)	15.7 ± 1.6	17.8 ± 4.6	0.051
Ganglion cell/inner plexiform layer (GCL/IPL)	51.5 ± 10.2	57.5 ± 15.7	0.244
Inner nuclear layer (INL)	29.8 ± 7.9	31.8 ± 7.7	0.515
Outer plexiform layer (OPL)	21.0 ± 3.0	23.1 ± 6.0	0.182
Outer nuclear layer (ONL)	83.3 ± 6.7	88.5 ± 17.4	0.253
Photoreceptor inner segments (IS)	33.0 ± 1.9	35.1 ± 7.6	0.208
Photoreceptor outer segments (OS)	41.7 ± 4.5	40.3 ± 10.4	0.599
Retinal pigment epithelium (RPE)	16.5 ± 2.7	19.9 ± 4.6	0.012*
Choriocapillaris (CC)	20.2 ± 4.9	19.6 ± 3.3	0.728
Outer choroid (C)	158.8 ± 23.2	173.2 ± 40.5	0.202
Total choroidal thickness	179.1 ± 25.0	192.8 ± 41.7	0.246
	**Soft Drusen (n = 12)**	**Hard Lesions (n = 11)**	**P-value**
Total retinal thickness	306.8 ± 15.4	321.8 ± 101.4	0.630
Nerve fiber layer (NFL)	16.7 ± 1.5	19.2 ± 6.3	0.202
Ganglion cell/inner plexiform layer (GCL/IPL)	55.1 ± 6.8	60.0 ± 21.9	0.487
Inner nuclear layer (INL)	31.1 ± 3.9	32.6 ± 10.6	0.667
Outer plexiform layer (OPL)	21.3 ± 2.8	25.0 ± 8.0	0.143
Outer nuclear layer (ONL)	90.8 ± 5.1	85.9 ± 24.9	0.528
Photoreceptor inner segments (IS)	33.6 ± 0.8	36.7 ± 11.0	0.354
Photoreceptor outer segments (OS)	38.5 ± 4.4	42.2 ± 14.4	0.415
Retinal pigment epithelium (RPE)	19.7 ± 2.9	20.1 ± 6.1	0.844
Choriocapillaris (CC)	19.9 ± 3.3	19.3 ± 3.5	0.663
Outer choroid (C)	167.2 ± 38.0	179.7 ± 44.0	0.503
Total choroidal thickness	187.2 ± 37.7	199.0 ± 46.7	0.537

^*^Statistically significant.

### Genotype of Animals with Drusenoid Lesions

Genetic polymorphisms in the ARMS2 and HTRA1 loci associated with drusenoid lesions in rhesus macaques have been independently confirmed in colony animals from the Oregon National Primate Research Center, the Caribbean Primate Research Center (CPRC), and the German Primate Center (Deutsche Primatenzentrum or DPZ) in Gottingen, the majority of which originated from the CPRC colony from Cayo Santiago^23–25^. In our cohort of animals, the high-risk allele in the ARMS2 locus (SNP position 10682 T > C) occurred at a higher frequency (70.7%) than previously reported at CPRC (20.3%) or DPZ (49.2%), with no significant difference between eyes with and without drusenoid lesions (P = 1.000; Table 4

**Table 4:** Frequencies of risk alleles in ARMS2 and HTRA1 associated with drusen in rhesus macaques.

	Genotype	Frequency		P-value
		Normal Eyes	Any Drusen	
ARMS2 SNP 10682	TT CT CC	4 19 22	1 9 10	1.000
HTRA1 SNP 17076	CC AC AA	43 0 2	18 2 0	0.176
		**Soft Drusen**	**Hard Lesions**	
ARMS2 SNP 10682	TT CT CC	1 3 4	0 3 6	0.793
HTRA1 SNP 17076	CC AC AA	7 1 0	9 0 0	0.471

Abbreviations: SNP, Single-nucleotide polymorphisms.

). The risk allele in HTRA1 (SNP position 17076 C > A) occurred at much lower frequency (4.6%) than the other colonies (21.0% to 60.0%), and also showed no association with presence of drusenoid lesions (P = 0.176). When comparing animals with the soft drusen and hard lesions, we found no difference in risk allele distribution in either the ARMS2 (P = 0.793) or HTRA1 (P = 0.471) loci. Our results suggest that the genetic factors previously found to confer increased risk of developing drusen in other rhesus colonies, may not contribute as strongly in our cohort of animals at CNPRC.

## Discussion

Nonhuman primates are the only mammals that spontaneously develop drusenoid lesions similar to those in humans, and thus have significant translational potential as an animal model for developing therapies for AMD. Ocular imaging technologies are often employed in macaques for preclinical testing of novel treatments or drugs, but rarely for understanding the pathophysiology of age-related retinal changes that mimic human disease. In this study, we employed validated measures of drusenoid lesions from fundus photographs, as well as a battery of ocular imaging modalities employed in human clinical studies, to provide the first robust, quantitative characterization of drusenoid lesions in rhesus macaques. We describe two distinct clinical phenotypes of “soft” drusen and “hard” punctate lesions that can be distinguished by their size on fundus photographs and appearance on SD-OCT. We also provide important normative macular thickness values of retinal and choroidal layers on SD-OCT, and found a slightly thicker RPE in macaque eyes with drusenoid lesions. Taken together, these data provide an important framework for research in drusen formation and AMD using rhesus macaques.

The AREDS2 system for grading AMD lesions is derived from major clinical trials sponsored by the National Eye Institute to study the effects of nutritional antioxidant supplements including lutein and zeaxanthin in humans with AMD. This validated system is highly reproducible and predictive of 5-year risk for progression to advanced AMD in humans, which is defined by the presence of center-involving RPE atrophy or neovascular AMD^36^. Interestingly, neither form of advanced AMD has ever been clearly documented in nonhuman primates, and we did not identify any cases in our survey of the rhesus colony at CNPRC. One explanation is that the occurrence is so rare that it has not yet been observed in animals under study. The drusen grading in most of our cohort was equivalent to an AREDS severity scale of 4, which in humans correspond to a rate of only 4.8% for progression to advanced AMD over 5 years^30^. Many of the animals in the study are near the end of their lifespan, and thus may not survive long enough for this progression to occur. Rhesus macaques also do not have the same genetic risk factors for AMD as humans. Among a selection of 42 human DNA markers associated with drusen, 24 have been identified in rhesus monkeys, of which only 7 were polymorphic^37^, and only the ARMS2/HTRA1 loci have been found to be associated with drusen in these animals^23–25^. In addition, our study animals are partially housed indoors (mean outdoor time 53.6 ± 34.0%), and may have a healthier diet and lower sun exposure compared to their free-ranging counterparts. Studies have shown that rhesus monkeys fed a diet lacking macular pigment carotenoids lutein and zeaxanthin are more susceptible to blue-light damage and may develop drusen at an earlier age^38,39^, suggesting that standard laboratory diets low in fat and rich in xanthophylls may limit drusen progression also. In fact, we found only a 30.7% prevalence of drusenoid lesions in our rhesus colony at CNPRC compared to the 47% noted from the same colony surveyed 22 years ago^20^, and up to rates as high as 74% at other sites^17^, despite surveying an older cohort of animals (Table 5). From an anatomic standpoint, macaques have very dark uveal pigmentation^40^, which in humans is protective against AMD^41^. None of the animals we examined demonstrated pigmentary changes in the macula, which are also strongly-associated with AMD progression in humans^30^. In contrast, macular pigmentary changes have been more frequently reported in free-ranging animal colonies with incidences of up to 32%^15,18^. Thus, developing nonhuman primate models with more advanced stages of AMD may require long-term dietary and environmental modifications that better represent human behavior. Interestingly, Japanese macaques (Macaca fuscata) have an early-onset form of drusen with a dominant mode of inheritance^42^. Cynomologus macaques (Macaca fascicularis) also develop drusen in an age-dependent manner with a potential link to hematologic markers^43^. Thus, the quantitative and imaging techniques used in our study may provide the necessary platform to study other nonhuman primate models of AMD in a more rigorous manner.

**Table 5:** Summary of published studies of drusen frequency in various primate research facilities.

	Location	Total Animals	Age Range	Drusen Frequency	Pigment Change
El Mofty *et al*. 1978^15^	CPRC (free-ranging)	105	1–21	30%	32%
Stafford *et al*. 1984^19^	Various (most indoor)	574	10–31	5.9%	NR
Engel *et al*. 1988^17^	CPRC (free-ranging)	29	13–27	74%	NR
Monaco *et al*. 1990^18^	Naval Aerospace Research Lab (indoor & free-ranging)	100	1–13	31%	10%
Hope *et al*. 1992^14^	CPRC (free-ranging)	246	1–30	57.7%	NR
Olin *et al*. 1995^20^	CRPRC & WRPRC (indoor)	62	20–33	47%	NR
Gouras *et al*. 2008^21^	NIH & ONPRC (indoor)	121	10–39	61%	NR
Current study 2017	CNPRC (indoor & outdoor)	61	19–30	30.7%	0%

Abbreviations: CPRC, Caribbean Primate Research Center; CRPRC, California Regional Primate Research Center; WRPRC, Wisconsin Regional Primate Research Center; NIH, National Institute of Health; ONPRC, Oregon National Primate Research Center; NR, not recorded.

Modern ocular imaging technologies such as SD-OCT and FAF provide new ways to characterize drusen phenotypes in humans and macaques. SD-OCT of soft drusen in rhesus monkeys appear as dome-shaped hyperreflective deposits under the RPE, which is very similar to those seen in AMD^44–47^. However, the soft drusen in macaques appear more uniform in morphology, and lack any reflective drusen substructures or hyperreflective foci in the overlying outer retina which are predictive of progression to geographic atrophy in humans^47,48^. We also did not find any reticular pseudodrusen, which are more strongly associated with geographic atrophy^49^, in any of the aged animals in our colony. The absence of these drusen abnormalities may further explain the absence of any advanced AMD phenotype in nonhuman primates. Although we noted extramacular drusen in a selection of animals, their association with AMD progression in humans remain unclear. Peripheral drusen are more frequently observed in AMD patients, but may not be associated with major genetic risk alleles for AMD such as CFH and ARMS2^50^. Further studies using ultrawide-field imaging may better characterize the role of extramacular drusen in rhesus macaques^51^.

In this study, we also described hard, punctate lesions in rhesus macaques that are undetectable on SD-OCT. We cannot exclude the possibility that the lesions are so small that they are below the detection threshold of SD-OCT, although this is unlikely since the imaging system has an axial resolution of 3.87 μm per pixel, a transverse resolution of 4.82 μm per pixel, and a line scan density of 51 μm between B-scans, compared to the large number of punctate lesions sampled which on average were approximately 10 μm in diameter on fundus photographs. Interestingly, similar small hypopigmented lesions have been described on histology in macaques as a form of lipoidal degeneration where RPE cells become filled with vacuoles and lipid droplets, rather than true sub-RPE deposits as seen with AMD drusen^52–54^. It is unclear, however, whether these punctate lesions represent a separate entity or are precursors to larger soft drusen. We noted that eyes with mostly soft drusen also have punctate lesions, but those with predominantly hard lesions rarely exhibit any soft drusen. Animals with soft drusen are also older than those with mainly hard drusen (25.2 ± 2.5 years vs. 22.3 ± 2.2 years; P = 0.024). Finally, while eyes with drusenoid lesions demonstrated RPE thickening, there was no significant difference in RPE thickness between animals with soft drusen or hard lesions. Together, these evidences suggest that the two drusen phenotypes may be different manifestations of the same pathophysiological process, and that hard, punctate lesions may occur at an earlier stage. This is further supported by the similar distribution of risk alleles in ARMS2 and HTRA1 between animals with the two drusen phenotypes. Since SD-OCT imaging affords the ability to monitor morphologic changes of these lesions over time, future longitudinal studies using SD-OCT and fundus photography may enhance our understanding of drusen progression in nonhuman primates.

FAF imaging provides another measure of disease activity in retinal conditions, although the precise source of the autofluorescence remains controversial^55–57^. Many hypothesize that blue-peak FAF is a measure of lipofuscin, autofluorescent pigment granules in RPE cells that result from lysosomal digestion of photoreceptor outer segments^58^. Rhesus monkeys deficient in lutein, zeaxanthin, and omega-3 fatty acids, for example, may exhibit elevated FAF intensities and are also associated with higher incidences of drusen lesions^59^. However, the FAF signature of macaque drusen have never been clearly explored. In humans, drusen may exhibit variable FAF patterns, likely due to differences in drusen size, composition, and effects on overlying RPE and photoreceptors^60,61^. In our study, different drusenoid lesions exhibit distinct levels of FAF, and may represent variable amounts and types of fluorophores within the lesions themselves or different stages of lipofuscin accumulation or RPE degeneration. Thus, including FAF imaging with longitudinal studies may further our insight into the pathophysiology of drusen development and RPE atrophy in these animals^62,63^.

Our study employs a systematic approach using multimodal ocular imaging to characterize and monitor drusenoid lesions in the aged rhesus colony at CNPRC. In contrast to prior reports from other primate centers where the prevalence of drusenoid lesions is higher, the current study is limited by the smaller cohort of affected animals at CNPRC. Compared to human subjects, ocular imaging of macaques is more challenging, with occasional ocular pathologies such as corneal scars or senile cataracts limiting our ability to attain high-resolution images in every animal. Also, while the AREDS2 grading system has been validated in human studies, the modified form calibrated from the anatomic dimensions of the macaque fundus still require further assessment. Nevertheless, using the tools developed in this research, future studies to characterize the progression of drusenoid lesions in rhesus macaques will be imperative toward establishing a powerful nonhuman primate model of AMD.

## Electronic supplementary material

Supplemental Table
