# Supplementary material for: In Vivo Multimodal Imaging of Drusenoid Lesions in Rhesus Macaques
Source: Sci Rep. 2017 Nov 3;7:15013. doi: 10.1038/s41598-017-14715-z (PMC5670133; doi:10.1038/s41598-017-14715-z)
Supplement: Supplementary file 1 — Supplemental Table [file 41598_2017_14715_MOESM1_ESM.doc]

**In Vivo Multimodal Imaging of Drusenoid Lesions in Rhesus Macaques**

Glenn Yiu, MD, PhD^1^, Eric Tieu^1^, Christian Munevar^2^, Brittany Wong^3^, David Cunefare^3^, Sina Farsiu, PhD^3^, Laura Garzel, DVM^4^, Jeffrey Roberts, DVM^2,4^, Sara M. Thomasy, DVM, PhD^1,2^

^1^Department of Ophthalmology & Vision Science, University of California, Davis, Sacramento, California

^2^Department of Surgical and Radiological Sciences, School of Veterinary Medicine, University of California, Davis, Davis, California

^3^Department of Biomedical Engineering, Duke University, Durham, North Carolina

^4^California National Primate Research Center, Davis, California

Corresponding author:

Glenn Yiu, MD, PhD

Department of Ophthalmology & Vision Science

University of California, Davis Eye Center

4860 Y St., Suite 2400

Sacramento, CA 95817

Phone: 916-734-6602

Email: [gyiu@ucdavis.edu](mailto:gyiu@ucdavis.edu)

**Supplemental Table 1. AREDS classification for grading digital fundus photographs**

| **Retinal Finding** | **Grading Codes & Regions Evaluated** |
| --- | --- |
| **Drusen size*** | 0: Absent  1: Questionable  2: Definite, diameter < C-0^&^ (≤58μm)  3: Diameter > C-0, but < C-1 (59-116μm)  4: Diameter > C-1, but < C-2 (117-233μm)  5: Diameter > C-2 (≥234μm)  8: Cannot grade |
| **Drusen area**^#^ | 0: Area < C-0  1: Area ≥ C-0, but < C-1  2: Area ≥ C-1, but < C-2  3: Area ≥ C-2, but < I-2  4: Area ≥ I-2, but < O-2  5: Area ≥ O-2, but < ½ DA  6: Area ≥ ½ DA, but < 1DA  7: Area ≥ 1DA  8: Cannot grade |
| **Drusen outside grid** | 0: None, questionable, or area < Circle O-2  5: Area ≥ O-2  8: Cannot grade |
| **Calcified drusen**  **Drusenoid pigment epithelial detachment**  **Reticular drusen** | 0: Absent  1: Questionable  2: Definite  8: Cannot grade |
| **Hyperpigmentation area**^#^ | 0: Absent  1: Questionable  2: Definite, area < C-2  3: Definite, area ≥ C-2  8: Cannot grade |
| **Hypopigmentation area**^#^ | 0: Absent  1: Questionable  2: Definite, area < C-2  3: Definite, area ≥ C-2  8: Cannot grade |
| **Geographic atrophy area**^#^ | disc areas by planimetry |
| **Geographic atrophy center involvement** | 0: Absent  1: Questionable  2: Definite  8: Cannot grade |
| **Neovascular AMD** | 0: Absent  1: Questionable  2: Definite  8: Cannot grade |

* Maximum size of drusen

^#^ Area within modified Early Treatment of Diabetic Retinopathy Study (ETDRS) grid

^&^ The circular areas for grading measured in square millimeters are: C-0 = 0.003 mm^2^, C-1 = 0.011 mm^2^, C-2 = 0.043 mm^2^, I-2 = 0.089 mm^2^, O-2 = 0.297 mm^2^, 1/2 disc area (DA) = 0.77 mm^2^, 1 DA = 1.54 mm^2^.
